# SARS Epidemic in the Press

**DOI:** 10.3201/eid1002.030743

**Published:** 2004-02

**Authors:** Giovanni Rezza, Raffaella Marino, Francesca Farchi, Mirella Taranto, Istituto Superiore di Sanità, Rome, Italy

**Affiliations:** * Istituto Superiore di Sanità, Rome, Italy

**To the Editor:** On March 12th, the World Health Organization *(*WHO) issued a global alert regarding severe acute respiratory syndrome (SARS) in Vietnam, Hong Kong, and China’s Guangdong Province. Three days later, for the first time in its history, WHO recommended postponing nonessential travel to the affected areas and screening airline passengers (*1*). These initiatives, together with the awareness of the modes transmission of the coronavirus associated with SARS (SARS-CoV), led to extensive press coverage.

To describe the extent of this coverage in Italy and to identify the events that prompted peak coverage, we reviewed the five Italian daily newspapers with the highest circulation (*3*) from March 12 to March 30. The articles were identified by hand search (reading headlines, subheads, and titles) and were classified according to the publication date and page number. We assigned one point to full articles and to front-page articles or headlines and half a point to short articles. We also reviewed all national newspapers for articles published before the travel advisory (March 12–15).

Before the travel advisory, no articles were published in the five newspapers, whereas on March 14, one article was published in a smaller newspaper (“Osservatore Romano”, the Vatican newspaper). On March 16 (the day after the advisory), six articles appeared in the five newspapers; through May 31, a total of 750 articles were published. The proportion of articles that appeared on the front-page was 9.6%, although this percentage was higher early in the study (50%) than at the time of absolute peak coverage (12%).

After the first wave of articles in mid-March, several peaks occurred until mid-April. The events prompting these peaks were identified by determining the most frequently covered topics, specifically: the death of Carlo Urbani, the Italian WHO officer who identified the disease in Hanoi; the first two probable cases in Italy; the death of a suspected case in Naples; and the press conference announcing the first meeting of the Italian National Task Force. The highest peak occurred on April 23, after the announcement that the number of cases had reached 4,000 and that a vaccine would not be available anytime soon. In the days after the peak, coverage remained quite high, in association with the definition of SARS as a “global threat” by WHO and the twofold increase in the number of probable cases in Italy. The high press coverage was followed by an overall decrease, although small peaks occurred in association with the conflicts among European Ministries on airport measures, increased quarantine measures in China, and the identification of the civet cat as a probable source of SARS-CoV. Coverage tended to be greater on weekends, probably because political stories constitute less competition for space on these days.

Evidently, the daily newspaper coverage of SARS has been quite extensive in Italy, especially in the aftermath of WHO alerts and statements by the Ministry of Health regarding new cases and more stringent control measures. During outbreaks of infections, both the media and the public are often criticized for overreacting, yet public concern over serious health hazards is essential in guiding prevention activities (*3*–*5*) and in deciding whether to adopt measures that could place restrictions on civil rights, such as quarantine (*6*). Although we did not evaluate the quality of risk-communication of the journalists or of the experts quoted in the articles, wide press coverage of the WHO global alert may have contributed to public-health bodies’ taking action towards containing the epidemic.

**Figure 1:**
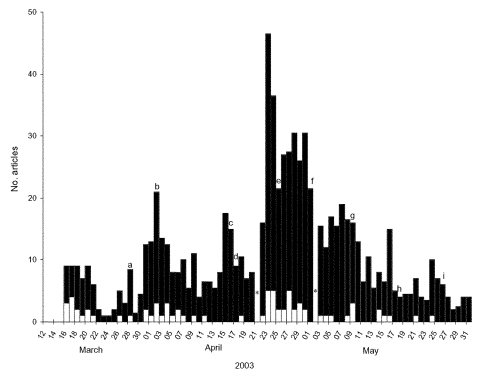
Number of articles on SARS published in the five newspapers with the highest nationwide circulation in Italy, by date of publication; March 15 to May 31, 2003. The white area of the bars represents the number of articles or headlines appearing on the front page. The peaks prompted by the specific events listed are indicated with arrows. An asterisk indicates days on which newspapers were not published (Easter and May 1). The World Health Organization (WHO) global alert was March 12 and the WHO travel advisory was March 15. a, death of Carlo Urbani; b, first 2 probable cases in Italy; c, task force press conference; d, death of suspected case in Naples; e, WHO warning of “global threat”; f, two-fold increase of probable cases in Italy; g, European conflict on airport measures; h, increased quarantine measures in China; i, Civet cat identified as probable source.
